# Do Not Forget the Stimulus: A Missing Control in Naturalistic Studies of Neural Entrainment

**DOI:** 10.1523/ENEURO.0451-25.2026

**Published:** 2026-06-24

**Authors:** Víctor J. López-Madrona, Jacques Pesnot Lerousseau, Benjamin Morillon

**Affiliations:** ^1^INSERM, INS, Institut de Neurosciences des Systèmes, Aix-Marseille Université, Marseille 13005, France; ^2^Institute of Language, Communication, and the Brain, Aix-Marseille Université, Marseille 13604, France

**Keywords:** auditory cortex, intracerebral recordings, language, speech perception

## Abstract

A main question in cognitive neuroscience is whether brain rhythms during speech perception reflect intrinsic neural oscillations or simply responses evoked by the acoustic signal. A recent intracranial study interpreted persistent auditory activity after acoustic edges as evidence for entrainment. We show that a similar rhythmic pattern is already present in the speech stimulus itself, supporting an evoked-response account instead. This highlights the need for routine stimulus–signal analyses as a critical control in studies of naturalistic speech processing.

A long-standing debate in cognitive neuroscience is whether brain rhythms observed during speech perception are actively generated by intrinsic neural oscillations (entrainment hypothesis) or whether they simply reflect evoked responses to the speech signal which is itself partly rhythmic (evoked hypothesis). In a recent paper, Akkol et al. (2025) analyzed intracranial recordings during naturalistic speech perception and reported that auditory cortical activity persisted for more than one cycle after acoustic edges, which they interpreted as evidence for neural entrainment. Here, we show that the same spectrotemporal pattern is present in the auditory stimulus itself, supporting an opposite interpretation consistent with the evoked hypothesis. We propose a good practice for future studies on naturalistic speech processing: the systematic analysis of the stimulus signal as a control condition.

In their study, Akkol et al. (2025) investigated brain activity in the auditory cortex during speech perception. The authors analyzed brain responses time-locked to the acoustic edges of the speech envelope, known to be acoustic landmarks (Oganian and Chang, 2019). They report that brain responses in the theta–alpha band last more than one oscillatory cycle—which the authors claim to be a hallmark of neural entrainment. They argue that “[t]his activity exceeded what could be expected from a simple evoked response,” suggesting that “speech has the potential to entrain neural oscillations in the human auditory cortex.”

However, we believe that the authors overlooked the possibility that the stimulus itself could be the source of such an effect. This needs to be investigated because speech is a complex waveform, and as such, it can exhibit nontrivial regularities. We replicated the analysis of Akkol and colleagues, but instead of using neural data, we analyzed their auditory stimulus—kindly provided by the authors. As illustrated in Figure 1, we computed the intertrial phase coherence (ITPC) time-locked to acoustic edges of the speech envelope, following the methodology described in Akkol et al. (2025).

**Figure 1.**
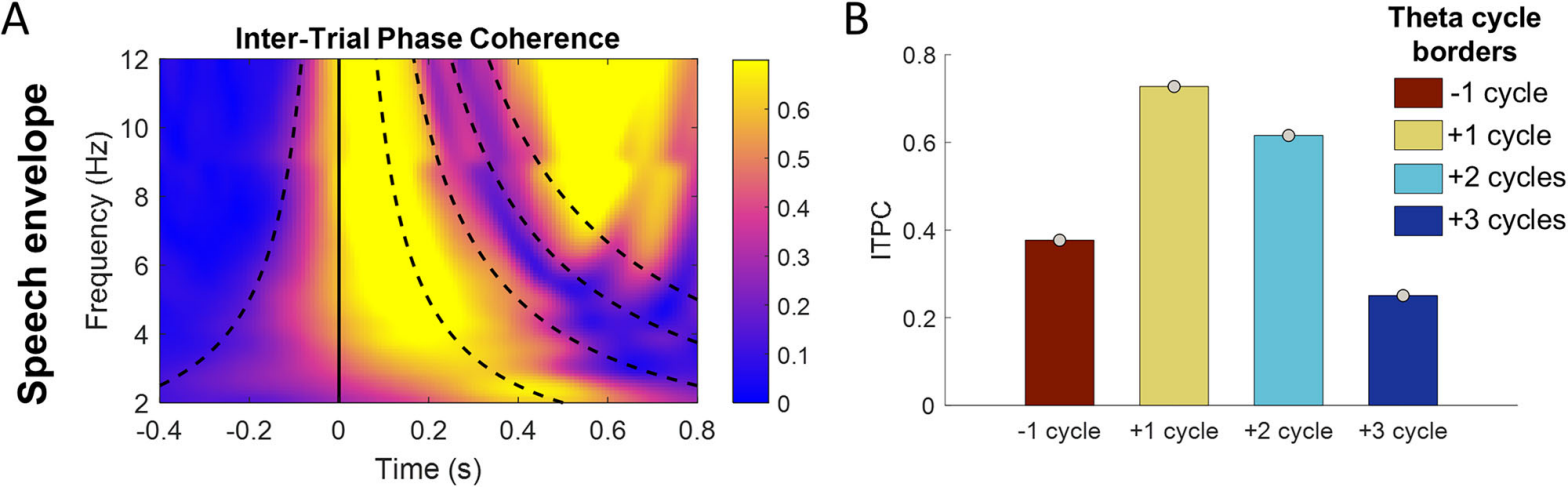
***A***, ITPC of the speech envelope time-locked to the acoustic edges. A −50 ms lag was applied to account for the speech tracking delay. ***B***, Averaged ITPC values in the theta band (4–8 Hz) at each cycle between the first pre-event cycle (−1) and third postevent cycle (+3).

The analysis used the speech envelope and acoustic edge markers provided by the authors. The speech envelope was computed using a cochlear-inspired filter bank (128 asymmetric filters log-spaced between 180 and 8,000 Hz), averaging Hilbert envelopes across filters. The envelope rate was the rectified first derivative of the envelope, smoothed with a 10 ms Gaussian kernel. Acoustic edges were defined as local maxima in the envelope rate (Oganian and Chang, 2019). Time–frequency decomposition used Morlet wavelets, with 67 log-spaced frequencies (1–200 Hz), 10 ms temporal resolution, and a frequency-dependent number of cycles (4 < 4 Hz, 5 for 4–9 Hz, 6 for 9–13 Hz; 7 > 13 Hz). To account for stimulus-to-brain positive latency (Xu et al., 2023), we applied a 50 ms time shift to the stimulus edge times (results were qualitatively similar for shifts between 20 and 200 ms).

Remarkably, applying a pipeline matched to Akkol et al. to the stimulus envelope yields a theta-band (4–8 Hz) ITPC pattern that closely matches the pattern reported in the neural data [Akkol et al. (2025), their Fig. 3], suggesting that much of the apparent complexity in the neural response may already be present in the input itself.

We propose to generalize this “good practice” for all studies on auditory perception and speech processing using naturalistic stimuli: the stimulus itself should be used as a control condition (López-Madrona et al., 2026). This issue is often acknowledged in the literature, but it is rarely examined in depth. For instance, Luo and Poeppel (2007) showed that theta-band phase activity can discriminate between speech sentences. They interpreted this as evidence for an intrinsic cortical rhythm operating at theta. It was not until a decade later that Ding et al. (2017) demonstrated that this theta activity corresponds to the main speech rate, whose acoustic peaks and edges are known to be the strongest drivers of auditory evoked responses (Oganian and Chang, 2019). Consistently, we recently demonstrated that the auditory theta response during naturalistic speech perception can be entirely explained away by the temporal dynamics of the speech envelope (López-Madrona et al., 2026). In line with this reasoning, Daube et al. (2019) further showed that cortical responses previously interpreted as phoneme-level representations can be explained by simple acoustic features of speech. These findings indicate that interpreting brain responses without fully considering the structure of the stimulus may lead to misleading conclusions.

Importantly, while stimulus-based controls can highlight the evoked nature of complex auditory responses to speech, the absence of a given pattern in the stimulus does not rule out an evoked neural origin. In such cases, explicit modeling of neural responses to acoustic features (incorporating response dynamics and noise) provides a quantitative estimate of the extent to which neural activity can be explained by evoked responses and to compare evoked and oscillatory accounts (Oganian et al., 2023).

While we do not question the empirical results of Akkol et al. (2025), our proposed approach offers an alternative interpretation that leans toward the evoked response rather than the entrainment hypothesis. Namely, complex rhythms in the early auditory cortex mirror speech acoustics (López-Madrona et al., 2026). Overall, we emphasize that while studying naturalistic stimuli opens exciting avenues for understanding cognition and neurophysiology, a careful analysis of the stimulus itself is a crucial first step. We advocate that such an analysis should become a standard good practice before interpreting neural dynamics.

## Synthesis

Reviewing Editor: Anne Keitel, University of Dundee Decisions are customarily a result of the Reviewing Editor and the peer reviewers coming together and discussing their recommendations until a consensus is reached. When revisions are invited, a fact-based synthesis statement explaining their decision and outlining what is needed to prepare a revision will be listed below. The following reviewer(s) agreed to reveal their identity: Yulia Oganian.

The manuscript has been reviewed by two expert reviewers as well as the editor. The reviewers consider the manuscript as a well-written, timely, and important commentary that highlights a significant methodological issue, though one reviewer notes that it reiterates a long-known point. Both reviewers recommend publication pending relatively minor revisions, mainly clarifying methodological details, crediting prior work, and elaborating on conceptual explanations.

Below are the synthesised reviewer comments, please respond to each in a point-by-point manner.

(Overall assessments for your information)

The manuscript points out a very important short-coming of the original paper and puts it in the context of a general methodological proposal for the field - to model not only the neural data but also the speech acoustics. This is a very well written and timely commentary. and I agree with all of its main points, but have two concerns that should be addressed before publication.

This manuscript makes an important point about a misinterpretation of results that has recently been published elsewhere. The authors demonstrate that a sustained inter-trial phase coherence measured at speech edges can not only be seen in neural, but also in the acoustic stimulus data. This shows that interpreting sustained ITPC in neural data as such does not prove an oscillatory tracking of speech. This manuscript is important, but ultimately just a reminder of what has long been known.

Reviewer comments - please respond to these

Conceptual points

1. The manuscript should credit and discuss the original paper on which the analyses of Akkol et al 2025 were based, namely Oganian, Kojima et al 2023. My understanding is that (among many other shortcomings of Akkol et al 2025), Akkol et al incompletely implemented the work by Oganian, Kojima et al, entirely ommitting the modeling aspect of that paper.

This is important because the evoked response model of Oganian et al. directly tests whether neural responses may have been due to the complex non-linear dynamics in the stimulus. While an analysis of the stimulus is a simple and straight-forward test, the model by Oganian et al. adds modeling of neural response dynamics and includes physiological noise, which can substantially change phase-locking dynamics observed neurally.

2. It should be made clear that even if a certain pattern is not visible in the stimulus, it may still be due to evoked neural activity.

3. Please clarify what is meant by "It remains possible, of course, that the proposed control analysis does not account for all aspects of the brain response. In that case, it would reinforce the hypothesis of genuine neural entrainment, [...]", e.g. with an example. I am not entirely sure I know what the authors mean here.

Methodological points

4. Please provide the details of how the envelope was extracted and processed to yield the ITPC data in figure 1 (merely referring to methods section of original article is in my view insufficient). In particular, how was the data filtered and/or transformed to TF-domain? Different choices could lead to more or less "ringing", so this is a crucial detail.

5. Please clarify this part: "and included a conservative -50 ms lag to account for the speech tracking delay (Xu et al., 2025). Qualitatively similar results were obtained for lags between -20 and 200 ms". What does -50 ms mean here? Why was this chosen? How does it relate to -20 - 200 ms?

## Author Response

Reviewer comments - please respond to these Conceptual points We thank the reviewers and the editor for their positive evaluation of the manuscript, as well as for their constructive comments. Below, we address their points in detail.

1. The manuscript should credit and discuss the original paper on which the analyses of Akkol et al 2025 were based, namely Oganian, Kojima et al 2023. My understanding is that (among many other shortcomings of Akkol et al 2025), Akkol et al incompletely implemented the work by Oganian, Kojima et al, entirely ommitting the modeling aspect of that paper.

This is important because the evoked response model of Oganian et al. directly tests whether neural responses may have been due to the complex non-linear dynamics in the stimulus. While an analysis of the stimulus is a simple and straight-forward test, the model by Oganian et al. adds modeling of neural response dynamics and includes physiological noise, which can substantially change phase-locking dynamics observed neurally.

We have added a new paragraph discussing this work and the added value of modeling approaches compared with our proposed control analysis: "Importantly, while stimulus-based controls can highlight the evoked nature of complex auditory responses to speech, the absence of a given pattern in the stimulus does not rule out an evoked neural origin. In such cases, explicit modelling of neural responses to acoustic features (incorporating response dynamics and noise), provides a quantitative estimate of the extent to which neural activity can be explained by evoked responses and to compare evoked and oscillatory accounts (Oganian et al., 2023).

2. It should be made clear that even if a certain pattern is not visible in the stimulus, it may still be due to evoked neural activity.

Indeed, we have now included this point in the discussion of why a modeling approach constitutes a more complete alternative. "Importantly, while stimulus-based controls can highlight the evoked nature of complex auditory responses to speech, the absence of a given pattern in the stimulus does not rule out an evoked neural origin." 3. Please clarify what is meant by "It remains possible, of course, that the proposed control analysis does not account for all aspects of the brain response. In that case, it would reinforce the hypothesis of genuine neural entrainment, [...]", e.g. with an example. I am not entirely sure I know what the authors mean here.

We agree that this sentence was unclear and potentially misleading. We therefore removed it; the underlying point is now addressed more explicitly in the new paragraph contrasting evoked-response and oscillatory models.

Methodological points 4. Please provide the details of how the envelope was extracted and processed to yield the ITPC data in figure 1 (merely referring to methods section of original article is in my view insufficient). In particular, how was the data filtered and/or transformed to TF-domain? Different choices could lead to more or less "ringing", so this is a crucial detail.

We have included this information in the text "The analysis used the speech envelope and acoustic edge markers provided by the authors. The speech envelope was computed using a cochlear-inspired filter bank (128 asymmetric filters log-spaced between 180 and 8000 Hz), averaging Hilbert envelopes across filters. The envelope rate was the rectified first derivative of the envelope, smoothed with a 10 ms Gaussian kernel. Acoustic edges were defined as local maxima in the envelope rate (Oganian and Chang, 2019). Time-frequency decomposition used Morlet wavelets, with 67 log-spaced frequencies (1-200 Hz), 10 ms temporal resolution, and a frequency-dependent number of cycles (4 < 4 Hz, 5 for 4-9 Hz, 6 for 9-13 Hz; 7 >13 Hz)." 5. Please clarify this part: "and included a conservative -50 ms lag to account for the speech tracking delay (Xu et al., 2025). Qualitatively similar results were obtained for lags between -20 and 200 ms". What does -50 ms mean here? Why was this chosen? How does it relate to -20 - 200 ms? This lag represents the delay between the stimulus and the brain response. It can be interpreted as the onset latency of the evoked response or, in the context of syllabic tracking, as the delay between the speech envelope and the brain's theta rhythm. Because there is no well-defined or universally accepted value for this latency (for example, Xu et al., 2023 report delays of approximately 50 ms for gamma and 100 ms for theta), we explored a range of values between 20 and 200 ms and obtained qualitatively similar results across this range. We therefore chose to present the results at 50 ms, as a compromise between shorter and longer delays and because this value closely matches the delays reported by Akkol et al. We have modified the manuscript accordingly to clarify this point: "To account for stimulus-to-brain positive latency (e.g., Xu et al., 2023), we applied a 50 ms time shift to the stimulus edge times (results were qualitatively similar for shifts between 20 and 200 ms)."
